# Emotional Dysregulation and Post-Traumatic Stress Symptoms: Which Interaction in Adolescents and Young Adults? A Systematic Review

**DOI:** 10.3390/brainsci13121730

**Published:** 2023-12-18

**Authors:** Lorenzo Conti, Sara Fantasia, Miriam Violi, Valerio Dell’Oste, Virginia Pedrinelli, Claudia Carmassi

**Affiliations:** Department of Clinical and Experimental Medicine, University of Pisa, 56126 Pisa, Italymiriamvioli@gmail.com (M.V.); virginiapedrinelli@gmail.com (V.P.); claudia.carmassi@unipi.it (C.C.)

**Keywords:** emotional dysregulation, emotion dysregulation, young adults, trauma, interpersonal trauma, PTSD

## Abstract

Emotional dysregulation (ED) has recently been conceptualized as a transnosographic entity in major mental disorders, and increasing evidence has suggested association between ED and post-traumatic stress symptoms (PTSS), though the nature of this association is unclear. The aim of the present review was to examine the possible interplay between ED and trauma exposure in the literature, as well as a possible role for the comorbidity of PTSD or PTSS in adolescents and young adults. In particular, we explored whether ED may represent a risk factor for PTSD or, conversely, a consequence of traumatic exposure. This systematic review was conducted according to PRISMA 2020 guidelines in three databases (PubMed, Scopus, and Embase). The 34 studies included showed a wide heterogeneity in terms of the populations selected and outcomes examined. Most studies used the Difficulties in Emotion Regulation Scale (DERS) and examined the relationship between ED, trauma, and psychopathological manifestations after the occurrence of trauma, with a focus on child abuse. Although current data in the literature are heterogeneous and inconclusive, this research highlights the role of ED as a mechanism that may mediate vulnerability to PTSD, but also as a predictor of severity and maintenance of typical, atypical, or associated PTSD symptoms, suggesting prevention programs for PTSD and other mental disorders should support the development of emotion regulation strategies.

## 1. Introduction

Scientific research has recently shown a growing interest in the concepts of emotion regulation and dysregulation, but their description is still controversial. Emotion regulation (ER) is defined as the set of processes by which each person assesses, suppresses, maintains, or modifies the intensity, frequency, or duration of emotional responses to exhibit appropriate social behavior or achieve goals [1]. It is related to the awareness, acceptance, and understanding of emotions and the adaptive use of strategies to modulate them and, therefore, depends on the individual response to the emotion rather than on the characteristics of the emotional experience [2].

In contrast, emotional dysregulation (ED) has been described as “an individual’s inability to exert some or all aspects of the normative processes involved in emotion regulation in such a way that they do not result in functioning significantly below their baseline” [3]. Specifically, ED is characterized by an inability to recognize and accept emotions and to choose effective strategies to cope with the resulting emotions, an inappropriate and excessive emotional response compared to social norms, an uncontrolled and rapid change in emotions, and an abnormal allocation of attention to emotional stimuli [4,5]. Three groups of theoretical models of emotion regulation have been identified [6,7] in the framework of the explorer process of ED development. The first group is “the temporal-based models”, which yield specific emotion regulation strategies for each stage of emotion generation, namely, situation selection and situation modification, attention deployment, cognitive change, and response modulation. The second group is “the strategy-based models” (e.g., identifying “adaptive” and “maladaptive” [8,9] experiential avoidance, rumination, worry), which employ emotion regulation strategies in light of their negative and positive relationships with psychopathological symptoms. The last group, “the ability-based models of emotional regulation” [2,10], captures dispositional abilities (e.g., ability to engage in goal-directed behavior when experiencing negative emotions, access to emotional regulation strategies perceived as effective) that are involved in different emotion regulation strategies and situations.

Recently, ED has been related to possible traumatic exposure [11,12]. In particular, traumatic events may be associated with various psychopathological manifestations, including mood disorders and anxiety [13,14,15,16]. The current literature shows that individuals who have been exposed to various traumatic events may develop a general lack of emotion regulation, regardless of the nature of the precipitating traumatic event. Consistently, ED has been conceptualized as a transnosographic entity in major mental disorders, including post-traumatic stress disorder (PTSD) [13,17]. The post-traumatic stress symptom (PTSS) pattern may be characterized by the predominance of fear-based emotional and behavioral symptoms, anhedonic or dysphoric mood and negative cognitions, arousal and reactive externalizing symptoms, and dissociative symptoms. Finally, a combination of these symptom patterns is also possible. In this regard, ED has been found to be associated with hypervigilance and attentional biases, hyperarousal, emotional numbing, and irritability, which are psychopathological elements of PTSD [18,19]. At the same time, fear, worry, intrusive thoughts, and avoidance, essential diagnostic elements in the assessment of PTSD, are considered in the theoretical description of the mechanism of emotion regulation, suggesting an interplay between this latter and even subthreshold forms of PTSD [20,21].

While the current literature data support the association between ED and PTSS, the nature of this association is unclear. Therefore, the aim of this review is to examine the possible interplay between ED and post-traumatic stress symptomatology, as reported in the available literature, focusing on adolescents and young adults. 

## 2. Methods

### 2.1. Literature Search

A systematic search was conducted from 1 December 2022 in accordance with the PRISMA 2020 guidelines [22] and using the electronic databases PubMed, Scopus, and Embase. The following search terms, without filters, restriction, or limits, were used to identify all potentially eligible records: (((emotion* dysregulation) OR (emotional dys?regulation[Text Word])) AND (((ptsd[MeSH Terms]) OR (post traumatic stress disorder[MeSH Terms]) OR (post traumatic stress symptomps[Text Word]))) AND ((interpersonal trauma[Text Word]) OR (trauma[Text Word])) AND ((((adolescent[MeSH Terms]) OR (young adult[MeSH Terms]) OR (adolesc*) OR (teen*) OR (youth*))))). All studies to 1 December 2022 were included in the databases search. Two independent reviewers (L.C., S.F.) screened papers for inclusion, and disagreements were resolved by discussion. S.F. and L.C. showed agreement on 89.3% of the studies included. The discrepancy that occurred in the categorization steps for the remaining 3 papers was discussed with C.C., and a consensus was reached to exclude these articles. 

### 2.2. Eligibility Criteria

The criteria used to include studies in this review were as follows:

1. Human studies;
2. Studies that included individuals aged >13 and <23 years;
3. Studies that adopted a validated scale to evaluate ED, PTSD, PTSS, or trauma;
4. Articles available in English.

Studies investigating ED in animal models were excluded. Studies in the form of reviews, case reports, and editorials were also excluded.

### 2.3. Quality Assessment

The quality of articles included was assessed by a standardized tool adapted from [23]. Furthermore, we used the Quality Assessment Tool for Observational Cohort and Cross-Sectional Studies (QATOCCSS) to assess the quality of the other type of study. Each study was scored as either “good”, “fair”, or “poor” (see Table 1). The quality assessment was performed by two independent reviewers (L.C. and S.F.), and a third reviewer (C.C.) cross-checked the quality assessment result. Disagreements were discussed and resolved with the research team. The degree of agreement between the independent authors was good; the authors agreed with the quality assessment of all included articles.

## 3. Results

**Figure 1:**
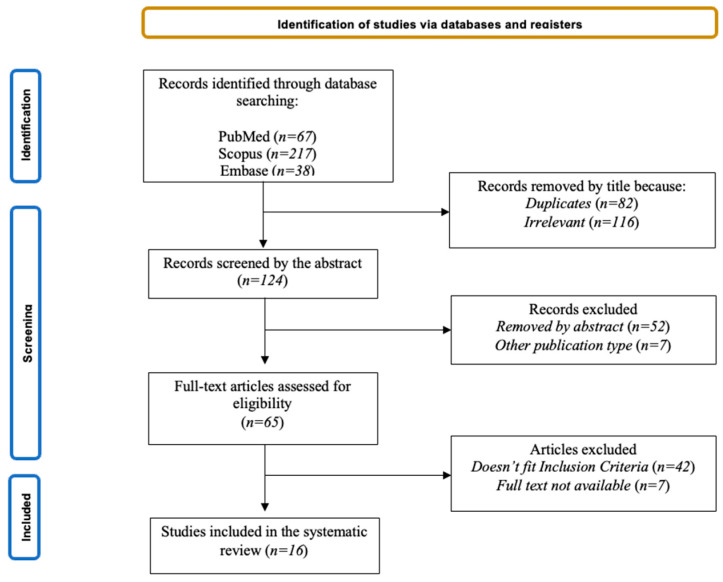
PRISMA flowchart of the study selection process. PRISMA, Preferred Reporting Items for Systematic Reviews and Meta-Analyses.

The primary databases search produced a total of 322 records. After that, 198 articles were removed based on titles because they were duplicates (*n* = 82) or not relevant (*n* = 116); 59 were removed based on abstracts because they were not pertinent (*n* = 52) or because they were other publication types (*n* = 7); *n* = 7 were removed because the full text was not available or not in English. After a full text reading, another 42 articles were excluded because they did not fit the eligibility criteria. Finally, a total of 16 articles were included in the present review, ranging from 2008 to 2022. We assessed the reference lists of selected papers for other eligible studies, and any disagreement on included papers was resolved via discussion. The grade of agreement between the two authors was good. Decisions for inclusion or exclusion are summarized in a flowchart according to PRISMA 2020 recommendations [22]. The study selection process is outlined in a flowchart (Figure 1). Details of each study included in the review are reported in Table 1.

### Emotional Dysregulation Assessment

To assess ED, 15 studies (93.75%) used one scale only. The most frequently adopted scale was the Difficulties in Emotion Regulation Scale (DERS) (*n* = 14; 87.50%); one study utilized this scale in combination with Paced Auditory Serial Addition Task—Computerized (PASAT-C). One study used the Affect Dysregulation (AD) and one used the Abbreviated Dysregulation Inventory (ADI).

To assess trauma exposure or PTSD, 12 studies (75.0%) used one scale only. The most utilized scale was the Posttraumatic Stress Disorder Checklist (PCL) (*n* = 6; 38.2%). The remaining articles heterogeneously used validated scales for trauma.

## 4. Discussion

In recent years, the scientific community’s growing interest in ED has given rise to its involvement in the field of PTSD. However, to the best of our knowledge, there is no systematic review analyzing the nature of this relationship. Moreover, the data available in the literature are particularly focused on the pediatric and young adult populations. Studies on emotional dysregulation in adults are present in the literature, but they are very heterogeneous. Therefore, the aim of this review is to clarify the interplay between ED and trauma exposure, particularly in their ED, in development or maintenance or as a possible clinical manifestation of PTSD in young adults.

The first data emerging from our work confirm that there is still no common and unambiguous definition of ED. This inevitably has implications for the interpretation and inclusion of clinical cases in studies. However, this heterogeneity seems to be partially overcome using the DERS as a method to assess ED in most of the studies included in this review. The DERS, developed and evaluated in an adult subject sample, is a self-report questionnaire divided into six domains: awareness—acceptance of emotions; clarity—knowledge of one’s emotions; goals—assessing difficulty with goal-directed behavior when angry; impulses—indicating difficulty with behavioral control when experiencing negative emotions; no acceptance—indicating negative secondary emotions; and strategies—the belief that no strategy can reduce negative emotions. 

In a few studies, the relationship between ED, trauma, and psychopathological manifestations was assessed ex post facto, that is, after the trauma occurred, with a frequent focus on childhood abuse. This result is related to current attachment theory, according to which the ability to regulate emotions develops in childhood through interactions with others, particularly the caregiver. Consequently, the presence of early traumatic interpersonal events interrupts this normal development [40,41,42]. 

This search highlights the role of ED as a mechanism that may mediate vulnerability to PTSD and act as a predictor of the severity and maintenance of typical, atypical, or associated symptoms of PTSD. Indeed, ED and the inability to engage in goal-directed behavior in the face of strong emotions related to a traumatic event may favor the development of PTSS and alcohol or substance use disorders or non-suicidal self-injury [27,43,44], corroborating the complex interplay between traumatic experiences and maladaptive copying in PTSD [45,46]. Adaptive cognitive styles (i.e., acceptance or positive reorientation and relativization) [47], on the other hand, may lead to greater resilience to symptoms of psychological distress, representing a note factor consistent with those already found in the literature [13,48,49].

In addition to PTSD and PTSS, the recent literature has emphasized the role of trauma, particularly childhood abuse, in other severe mental disorders such as treatment-resistant depression and especially borderline personality disorder (BPD), in which the role of neurodevelopmental traits such as being on the autism spectrum has been highlighted [50,51,52,53,54,55,56]. This review confirms the association between BP and ED and indicates greater emotional dysregulation in subjects with BP who were abused in childhood, as well as the role of the mediator of under-regulation of affect between childhood trauma and BP severity [57].

However, the “ex post facto” assessment of the included studies does not allow us to clarify whether ED is an entity that precedes the trauma, increasing the risk of exposure to the event as well as influencing its outcome, or whether it is a consequence of the trauma. 

Moreover, when discussing our results, some limitations should be kept in mind. First, we considered only English-language articles in our selection so there is a risk that relevant articles were overlooked. Second, the included studies show considerable heterogeneity in terms of methodology and results. Third, we did not take into account genetic predisposition of ED, but future studies should further investigate the correlation. Fourth, previous or subsequent treatments are not always specified because the proposed treatments are extremely heterogeneous, and often treatments are only observed in clinical cases.

## 5. Conclusions

The findings of this critical review suggest a possible role for ED which may represent a key mechanism in the development and maintenance of PTSD and PTSS in adolescents and young adults. They suggest that prevention programs for PTSD and mental disorders in general should include the development of emotion regulation strategies. However, as this work has shown, the data currently available in the literature are inconclusive and do not allow us to determine with certainty the nature of the relationship between ED and PTSD in adults. This review provides useful insights for future research on ED. It highlights the need for longitudinal studies involving subjects prior to trauma or those who have been exposed to trauma in adulthood.

## Figures and Tables

**Table 1:** Characteristics of the studies included in the systematic review. PTSD: post-traumatic stress disease; DEP: depression; IPV: intimate partner violence; ED: emotional dysregulation; RSB: risky sexual behavior; NSSI: non-suicidal self-injury; CSA: child sexual abuse; ASA: adult sexual abuse.

Study	Date	Country	Quality Rating	Sample Size	Population	Mean Age	ED Scale	Trauma/PTSD Scale	Another Psychopathological Scale	Main Findings
Marsee et al. [24]	2008	USA	Good	166	61% Female and 3% Caucasian	14.97	ADI	HURTE	PCS RI	Specific types of aggressive responses, particularly those that involve poorly regulated emotion, thus may be especially common following a traumatic event such as a hurricane.
Lilly et al. [25]	2013	USA	Good	404	Undergraduate: *n* = 290; and IPV: *n* = 114	22.37	DERS	PDS TLEQ	ECR-R WAS SOM DEP	Disruption in the ability to regulate emotions is the most consistent predictor of mental health in survivors of interpersonal trauma.
Valdez et al. [26]	2014	USA	Good	406	Female: *n* = 228; Male: *n* = 178	16.99	AD	DIS-IV	D-ARK	Emotion dysregulation was a stronger predictor of depressive symptoms for females than males.
Tull et al. [27]	2015	USA	Fair	151	100% Women *n* = 106; 76.7% African American; 21.4% White; 2.8% Multiracial; 1.9% Latina	21.9	DERS PASAT-C	LEC PCL	PANAS-NA DUQ	Emotion dysregulation may increase risk for substance use among women experiencing PTS symptoms.
Bennett et al. [28]	2015	USA	Good	225	Female: *n* = 83; Male: *n* = 142	16.23	DERS	PTSD-RI	PDEQ-C A-DES ENS	The presence of peritraumatic dissociation at the time of trauma may contribute to the continuation of dissociative symptoms as a more generalized pattern.
Tripp et al. [29]	2015	USA	Good	240	Female: *n* = 168	21.43	DERS	PCL-S	DDQ PANAS	Gender-specific interventions targeting emotion dysregulation may be effective in reducing alcohol-related consequences in individuals with PTSD.
Chaplo et al. [30]	2015	USA	Good	525	Male: *n* = 392; Female *n* = 133	16.11	DERS	PTSD-RI-AV	A-DES SASI-C	Dissociation and emotion dysregulation play in the relation between sexual abuse and self-injury.
Klanecky et al. [31]	2016	USA	Good	213	Male: *n* = 135; Female *n* = 78	19.56	DERS	ETI-SR-SF PCL-C	AUDIT DES-II	Drinking increased as dissociative tendencies increased and relations between the vulnerability factors (including emotional dysregulation).
Modrowski and Kerig [32]	2017	USA	Good	842	Female: *n* = 219; Male: *n* = 623	16.10	DERS	UCLA PTSD-RI	BBTS PDEQ-C	Dissociative subtype (with ED) membership may be more common in youth, particularly among those youths involved in the justice system.
Viana et al. [33]	2018	USA	Fair	74	52.0% Female	15.1	DERS	CPSS	CASI	Difficulties recognizing and accurately understanding emotions may increase risk for PTSD symptoms among trauma-exposed youth.
Charak et al. [34]	2018	USA	Good	809	MA: *n* = 327; VE: *n* = 337; PV: *n* = 145	16.08	DERS	PTSD-RI	MAY SI-2	Most justice-involved youths have experienced substantial adversity and evidence the most severe problems in emotion dysregulation and PTSD, internalizing and externalizing symptoms.
Weiss et al. [35]	2019	USA	Good	491	100% Women; 60.0% White; 36.2% Black/African American; 5.6% Latina; 4.5% American Indian; 4.3% Asian; 2.7% Other	21.80	DERS	LEC PCL	RSB	Provide support for the mediating roles of nonacceptance of negative emotions and difficulties controlling behaviors when distressed in the relation between PTSD symptoms and later RSB.
Trent et al. [36]	2019	USA	Fair	50	52.0% Female	15.5	DERS	CPSS	PTI CDI	Exposure to maternal threatening behaviors is related to more severe depressive symptoms among children with greater deficits in emotional clarity.
Raudales et al. [37]	2020	USA	Fair	60	Young Adult: *n* = 32; Undergraduate Research: *n* = 28	20.52	DERS	PCL-5	SCID-5 RSDI PANAS-NA	Emotion dysregulation is one mechanism which may confer vulnerability to PTSD symptomology.
Walker et al. [38]	2021	USA	Good	567	81.1% Female; 56.6% White	20.84	DERS	PCL-5	LSC-R DAR-5	CSA and ASA increase several factors that may be linked with increased risk for sexual victimization.
Andersson et al. [39]	2022	Sweden	Good	3169	43.6% Male; 55.6% Female	18.10	DERS	TSCC	SITBI	Increased levels of emotional dysregulation and trauma symptoms in relation to childhood abuse contribute to the increased risk of NSSI.

## Data Availability

All data generated or analyzed during this study are included in this published article.
